# Prognostic values of microRNA-130 family expression in patients with cancer: a meta-analysis and database test

**DOI:** 10.1186/s12967-019-2093-y

**Published:** 2019-10-22

**Authors:** Zhen Peng, Fujiao Duan, Jingjing Yin, Yajing Feng, Zhongyu Yang, Jia Shang

**Affiliations:** 1 Department of Infectious Disease, Henan Provincial People’s Hospital, People’s Hospital of Zhengzhou University, Zhengzhou, Henan 450003 China; 2 Medical Research Office, Affiliated Cancer Hospital of Zhengzhou University, Zhengzhou, Henan China; 3 College of Public Health, Zhengzhou University, Zhengzhou, Henan China; 4 Department of Nosocomial Infection Management, The First Affiliated Hospital of Zhengzhou University, Zhengzhou, Henan China; 5 College of Art and Science, The Ohio State University, Columbus, OH USA

**Keywords:** miRNA-130a, miRNA-130b, Cancer, Prognosis, Systematic evaluation

## Abstract

**Background:**

Emerging evidence shows that microRNA-130 (miRNA-130) family may be useful as prognostic biomarkers in cancer. However, there is no confirmation in an independent validation study. The aim of this study was to summarize the prognostic value of miRNA-130 family (miRNA-130a and miRNA-130b) for survival in patients with cancer.

**Methods:**

The pooled hazard ratios (HRs) with 95% confidence intervals (CIs) were calculated to estimate the association strength between miRNA-130 family expression and prognosis. Kaplan–Meier plotters were used to verify the miRNA-130b expression and overall survival (OS).

**Results:**

A total of 2141 patients with OS and 1159 patients with disease-free survival (DFS)/progression-free survival (PFS) were analyzed in evidence synthesis. For the miRNA-130a, the overall pooled effect size (HR) was HR 1.58 (95% CI: 1.21–2.06, *P* < 0.001). Tissue and serum expression of miRNA-130a was significantly associated with the OS (HR = 1.54, 95% CI: 1.11–2.14, *P *= 0.009; HR = 1.65, 95% CI: 1.14–2.38, *P *= 0.008), and in gastric cancer (HR = 1.81, 95% CI: 1.34–2.45, *P *< 0.001). For the miRNA-13b, a statistical correlation was observed between high miRNA-130b expression and poor OS in patients with cancer (HR = 1.95, 95% CI: 1.47–2.59, *P *< 0.001), especially in tissue sample (HR = 2.01, 95% CI: 1.39–2.91, *P *< 0.001), Asian (HR = 2.55, 95% Cl: 1.77–3.69, *P *< 0.001) and hepatocellular carcinoma (HR = 1.87, 95% CI: 1.23–2.85, *P *= 0.004). The expression of miRNA-130b was significantly correlated with DFS/PFS (HR = 1.53, 95% CI: 1.31–1.77, *P *< 0.001), in tissue (HR = 1.98, 95% CI: 1.50–2.62, *P *< 0.001) and serum (HR = 1.37, 95% CI: 1.15–1.64, *P *< 0.001), especially in HCC (HR = 1.98, 95% CI: 1.50, 2.62, *P *< 0.001). In database test, a significant correlation between high miRNA-130b expression and poor OS for HCC patients was observed (HR = 1.55, 95% CI: 1.01, 2.35, *P *= 0.0045).

**Conclusion:**

The high expression of miRNA-130 family might predict poor prognosis in cancer patients. Prospectively, combining miRNA-130a and miRNA-130b may be considered as powerful prognostic predictor for clinical application.

## Background

Cancer is recognized as the leading cause of human death [1], and the most important single barrier to improve life expectancy globally, in spite of advances in diagnosis and therapy [2]. Although cumulative tumor markers and pathological parameters have been discovered [3], only a few biomarkers can be used clinically because of tumor heterogeneity and biomarker extensibility [4]. And more effective and reliable biomarkers for cancer prognosis still need to be identified.

MicroRNA (miRNA) is a small (21 to 23 nucleotide), highly conserved, non-coding RNA that regulates gene expression by base pairing with the 3′-untranslated region (3′-UTR) of the mRNA [5]. Numerous studies have shown that miRNAs play crucial roles in biological behavior of various tumors, including proliferation, metastasis and invasion [6, 7]. Therefore, as non-invasive biomarkers, miRNAs may imply significant predictive value for tumor prognosis [8].

The miRNA-130 family, including miRNA-130a and miRNA-130b, is entangled intricately in tumor via an ambiguous way [9]. Studies have showed that miRNA-130a facilitated proliferation, invasion and metastasis of tumor cells [10–13], which may be associated with treatment resistance [14] and poor disease-free survival (DFS) [15] and overall survival (OS) [16, 17]. The potential mechanisms may involved down-regulation of tumor suppressor gene, phosphatase and tensin homolog (*PTEN*) in osteosarcoma and breast cancer [11, 12], runt-related transcription factor 3 (*RUNX*-*3*) and collapsing-response mediator protein type 4 (*CRMP4*) in gastric cancer [10, 17], peroxisome proliferator-activated receptor gamma (*PPARG*) in chorangiocarcinoma [16] and enhanced mammalian target of rapamycin (mTOR) signaling pathway in ovarian cancer [13]. However, a retrospective analysis showed that miRNA-130a had no significant effect on the prognosis of gastric cancer [18]. Moreover, miRNA-130a, acted like a tumor suppressor, has been reported to inhibit the androgen receptor (AR) and mitogen activated protein kinase (MAPK) pathways and target FOS-like antigen 1 (*FOSL1*) in prostate cancer [19], and triple-negative breast cancer [20], respectively. And an analysis of hepatocellular carcinoma (HCC) survival data suggested that high expression of miRNA-130a was significantly associated with long OS [21].

Studies have shown that epithelial–mesenchymal transition (EMT) could be induced by miRNA-130b in gliomas colorectal cancer and HCC via debilitation of *PPARG* [22] or *PTEN* [23] assisting malignant proliferation, metastasis and invasion, which may be associated with tumor progression and poor prognosis [24–26]. This characteristic that contributes to tumorigenesis and progression has also been discovered in gastric cancer, which may be achieved by mediation of *RUNX3* [27] and Ras-related protein activator like 1 (*RASAL1*) [28]. Nevertheless, according to their research, some scholars argued that miRNA-130b was not associated with tumor progression and OS/DFS in gliomas [29] and colorectal cancer [30]. Oppositely, miRNA-130b may attenuate the proliferation and invasion of pancreatic cancer cells by inhibiting the expression of signal transductor and activator of transcription 3 (*STAT3*) [31].

Based on the above controversial results, the prognostic significance of miRNA-130 family in tumors remains equivocal. Currently, there is insufficient support on evidence-based medicine for prognostic significance of miR-130 family in cancers, and the clinical application of targeting miRNA-130 family has also not been established yet. Therefore, we conducted this meta-analysis to further clarify the prognostic role of the miR-130 family in cancer, and miRNA-130 family expression in cancer may act as a prognostic predictor and potential therapy target in the future.

## Materials and methods

This study was conducted based on the guidelines of the meta-analysis of Observational Studies in Epidemiology (MOOSE) [32], and the Preferred Reporting Items for Systematic Reviews and Meta-Analysis (PRISMA) guidelines [33]. In the process of constructing the prognostic value of cancer related miRNA-130 family, we rely on the help of the population, interventions, comparators, outcomes and study designs (PICOS) principle to complete the research design.

### Search strategy

We conducted a systematic literature search until July 17, 2019 using Web of Science, PubMed, EMBASE, Cochrane Library, Wanfang (Chinese) and CNKI (Chinese) database. The combination terms “cancer” or “tumor” or “carcinoma” and “miRNA-130a or miRNA-130b or miRNA-130 family” and “survival” or “prognosis” or “outcome”. We also manually retrieved bibliographies of related studies that were not retrieved in the database.

### Inclusion and exclusion criteria

Inclusive criteria: (1) associations of expression of miRNA-130 family in cancer with OS, DFS and PFS or estimation of other survival probabilities were described, (2) patients with cancer were categorized into two groups based on high and low expression of miRNA-130 family, (3) hazard ratios (HR) with 95% CI for survival analysis were presented or could be reckoned from the instance data, (4) available in Chinese or English language. Exclusive criteria: (1) letters, reviews, expert opinions and case reports, (2) articles no available data for calculating HR with 95% CI, (3) duplicate publications.

If a study overlaps data from other published literatures, we choose to publish the latest one and/or the largest sample size.

### Data extraction

Data extraction following items were extracted from the eligible studies: The name of first author, publication year, patient ages and genders, follow-up duration, sample size, pathology subtypes, clinicopathological features, OS, DFS or PFS and HR with 95% CIs. All of the information was considered as independent data sets. If HR and 95% CI were not reported, the approach of Parmar [34] and Tierney [35] was used to extrapolate the HR with 95% CI.

### Methodological quality assessment

The methodological quality of eligible studies was evaluated by Newcastle-Ottawa Scale (NOS). NOS consists of three parts with a total of 9 points. Studies with NOS scores ≥ 6 points were considered as high-quality.

The specific Quality In Prognosis Studies (QUIPS) was assessed according to the method of Hayden et al. [36]. Estimates of potential bias include study participation, study attrition, prognostic factor measurement, outcome measurement, study confounding, statistical analysis, and reporting.

### miRNA-130b expression profile and prognosis

The KM plotter is a pooled analysis based on biomarker evaluation, which was used to measure the effect of miRNA-130b expression levels on OS of 372 HCC patients [37].

### Statistical analysis

Statistical analyses of pooled HRs with 95% CIs were conducted by Review Manager 5.3.5 (Cochrane Collaboration, Oxford, UK) to assess the association between miRNA-130 family expression and prognosis. Indicators of inter-study heterogeneity were tested by the Q-tests and *I*-squared (*I*^2^) [38]. Based on the results of heterogeneity analysis, when *P* value of heterogeneity (*P*_heterogeneity_) ≥ 0.10 or *I*^2^ ≤ 50%, a fixed-effects model (Mantel–Haenszel method) [39] was applied to calculate the pooled effect size, otherwise (*P*_heterogeneity_ < 0.1 and *I*^2^ > 50%) the random-effects model (DerSimonian and Laird method) [40] was employed, and the sources of heterogeneity was explored by meta-regression in STATA 13.1MP (StataCorp, College Station, TX, USA) [41]. In some of the articles that do not provide HR and 95% CI, Engauge Digitizer 10.0 (https://sourceforge.net/projects/digitizer/) was utilized to extract the original survival data from the KM curves. Subgroup analyses were performed by Prognostic indicators, ethnicity (Asian, Caucasian) and cancer subtypes (pathological type). Begg’s (rank correlation test) [42] and Egger’s test (weighted linear regression test) [43] were used to evaluate the extent of publication bias.

If the 95% CI did not overlap 1, and the pooled effect size estimate of HR > 1, they would be considered statistically significant. The KM plotter split was median, and all *P*-values were two-sided and *P* < 0.05 was considered statistically significant.

## Results

### Study identification

**Fig. 1:**
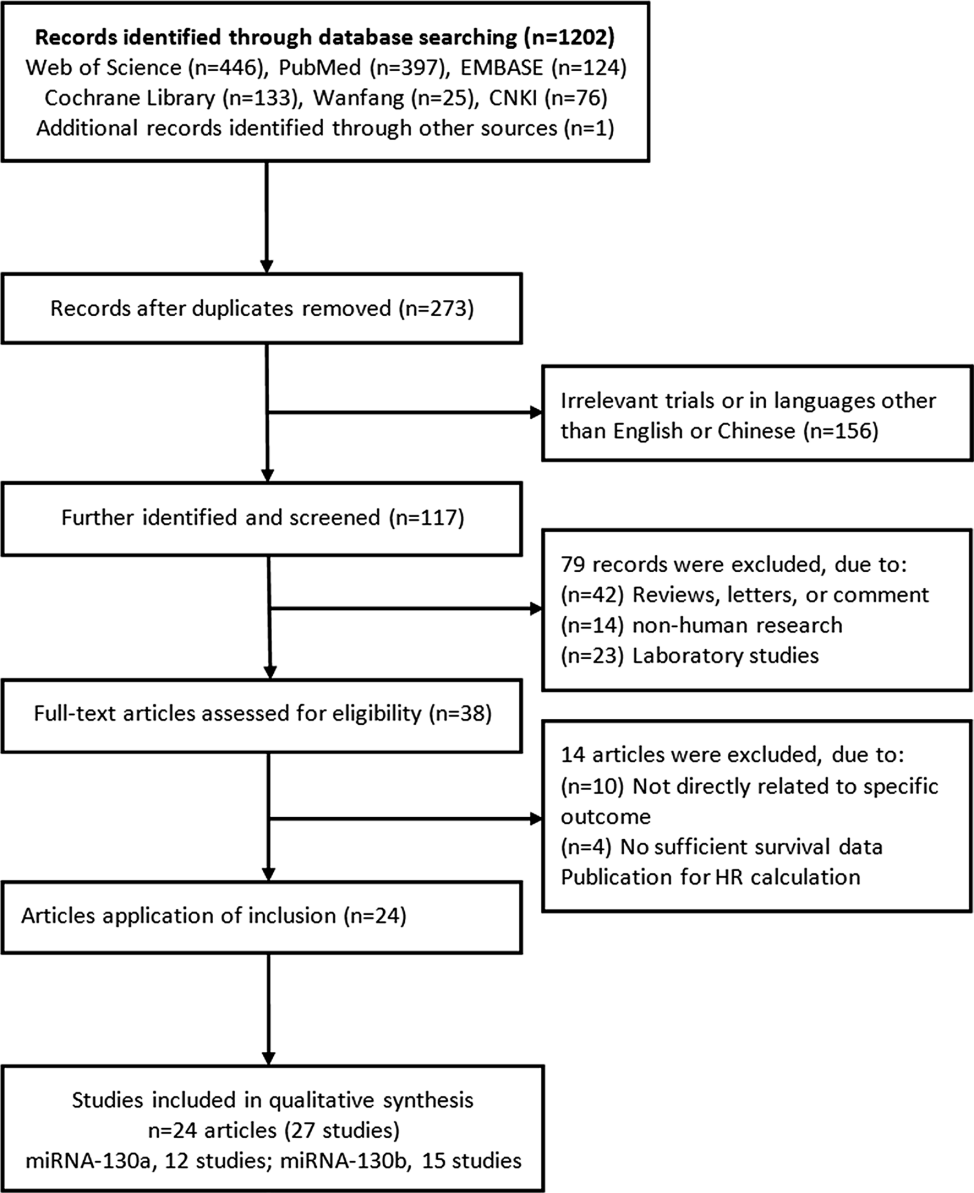
Flow chart of literature search and study selection

As shown in Fig. 1, a total of 1202 records were retrieved in the databases based on the search strategy. By screening the title and abstract, we excluded 929 duplicates and 156 unrelated records or language was neither English nor Chinese, and then retrieved 38 relevant full-text articles. Thirteen articles were further removed because no survival analysis results were reported and insufficient survival data were available to recalculate HR and 95% CI. Finally, 24 eligible articles (27 studies) [11, 14–18, 21–26, 30, 31, 44–53] including 12 articles [11, 14–18, 21, 44–48] for miRNA-130a and 12 articles (15 studies) [22–26, 30, 31, 49–53] for miRNA-130b were included in this meta-analysis. Three articles [23, 49, 52] included two cohort studies from different populations (Table 1).

**Table 1:** Clinicopathological characteristics of eligible studies

Study [Ref.]	Country	miRNA-130a		miRNA-130b		Histology	TNM stage	Sample	Assay	Follow-up (months)	Cut-off	HR (95% CI)	
		OS	Other	OS	Other							OS	DFS/PFS
Jia 2019 [44]	China	284				Gastric cancer	I–IV	Frozen tissue	qRT-PCR	50	Median	2.44 (1.35,4.40)	
Peng 2018 [18]	China	333	DFS,333			Gastric cancer	I–III	Serum	qRT-PCR	59	Median	1.49 (0.99,2.26)	1.38 (0.99,1.91)
Liu 2018 [45]	China	369				Colorectal cancer	I–IV	Serum	qRT-PCR	60	Median	2.36 (1.07,5.22)	
Yang 2018 [46]	China	60	DFS,60			Colorectal cancer	I–IV	Frozen tissue	qRT-PCR	70	Median	2.25 (1.05,4.83)	
Asukai 2017 [16]	Japan	27	DFS,27			Cholangiocarcinoma	NA	Frozen tissue	qRT-PCR	82	Median	2.36 (1.18,4.17)	2.47 (1.10,5.56)
Zhou 2017 [47]	China	51				HCC	I–III	Frozen tissue	qRT-PCR	42	Normal	1.23 (0.78,1.96)	
Chen 2016 [11]	China	86	DFS,86			Osteosarcoma	I–IV	Frozen tissue	qRT-PCR	60	Median	2.14 (1.14,4.02)	2.04 (1.22,3.40)
Jiang 2016 [17]	China	41				Gastric cancer	I–III	Frozen tissue	qRT-PCR	36	Normal	2.05 (1.03,4.08)	
Yuan 2016 [14]	China	56				Lymphoma	NA	Frozen tissue	qRT-PCR	50	Median	1.23 (0.94,1.61)	
He 2014 [15]	China	73	DFS,73			Cervical cancer	I–IV	Frozen tissue	qRT-PCR	86	Normal	1.41 (0.30,6.63)	1.73 (0.14,21.52)
Li 2014 [21]	China	102				HCC	I–III	Frozen tissue	qRT-PCR	72	Normal	0.45 (0.22,0.90)	
Wang 2012 [48]	China		DFS,100			NSCLC	I–III	Frozen tissue	qRT-PCR	96	Normal		0.21 (0.09,0.50)
Hashimoto 2019 [49]	America (AA)			36		Prostate cancer	I–IV	Frozen tissue	qRT-PCR	260	Mean	22.4 (2.27,221.3)	
	America (EA)			57		Prostate cancer	I–IV	Frozen tissue	qRT-PCR	250	Mean	1.10 (0.21,5.74)	
Ulivi 2019 [50]	Italy			83	DFS,85	NSCLC	I–IIIA	Serum	qRT-PCR	160	Median	1.35 (1.08,1.69)	1.35 (1.08,1.69)
Hu 2018 [51]	China			110		HCC	NA	Serum	qRT-PCR	60	Median	6.58 (3.04,14.24)	
Ecke 2017 [52]	Germany (TC)			100		Bladder-cancer	NA	Frozen tissue	qRT-PCR	156	Median	0.99 (0.82,1.20)	
	Germany (VC)			56		Bladder-cancer	NA	Frozen tissue	qRT-PCR	156	Median	1.02 (0.53,1.96)	
Li 2017 [22]	China			85		Glioma	NA	Frozen tissue	qRT-PCR	36	Mean	2.22(1.38,3.56)	
Chang 2016 [23]	China (TC)			85	DFS,85	HCC	I–III	Frozen tissue	qRT-PCR	66	Normal	1.01 (0.38,2.72)	2.02 (1.33,3.07)
	China (VC)			65	DFS,65	HCC	I–III	Frozen tissue	qRT-PCR	80	Normal	1.93 (1.30,2.87)	1.73 (1.16,2.58)
Sheng 2015 [24]	China			86		Glioma	NA	Frozen tissue	qRT-PCR	36	Median	4.39 (1.50,12.82)	
Wang 2014 [25]	China			97	DFS,97	HCC	I–IV	Frozen tissue	qRT-PCR	60	Median	2.52 (1.24,5.15)	4.00 (1.52,10.54)
Kjersem 2014 [30]	Norway			150	PFS,150	Colorectal cancer	NA	Serum	qRT-PCR	NA	Median	1.31 (0.96,1.79)	1.40 (1.05,1.86)
Colangelo 2013 [26]	Italy			80		Colorectal Cancer	I–IV	Frozen tissue	qRT-PCR	108	Mean	5.99 (1.99,18.03)	
Zhao 2013 [31]	China			52		Pancreatic cancer	I–IV	Frozen tissue	qRT-PCR	36	Median	2.84 (1.25,6.45)	
Nakatani 2012 [53]	Italy			49		Sarcoma	NA	Frozen tissue	qRT-PCR	217	Median	2.11 (1.22,3.63)	

NSCLC, non-small cell lung cancer; HCC, hepatocellular cancer; qRT-PCR, quantitative real-time PCR; OS, overall survival; PFS, progressive free survival; DFS, disease free survival; SC, survival curve; AA, African-American; EA, European-American; TC,Training cohort; VC, Validation cohort

### Baseline characteristics of eligible studies

The basic characteristics of included studies are presented in Table 1. The articles were published from 2012 to 2019 and a total of 2141 patients with OS and 1159 patients with DFS/PFS from China, Japan, America, Germany, Norway, and Italy. The country of the study was determined based on the regional source of the subjects. The types of cancer included gastric cancer, colorectal cancer, HCC, glioma, cholangiocarcinoma, osteosarcoma, lymphoma, non-small cell lung cancer, cervical cancer, pancreatic cancer and sarcoma. The method of all miRNA-130a and miRNA-130b detection was quantitative real-time polymerase chain reaction (qRT-PCR). The expression of miRNA-130 family for OS and/or DFS/PFS was measured in tissue or serum. The cut-off value of miRNA-130 family expression was mostly set by median.

### Assessment of methodological quality

**Fig. 2:**
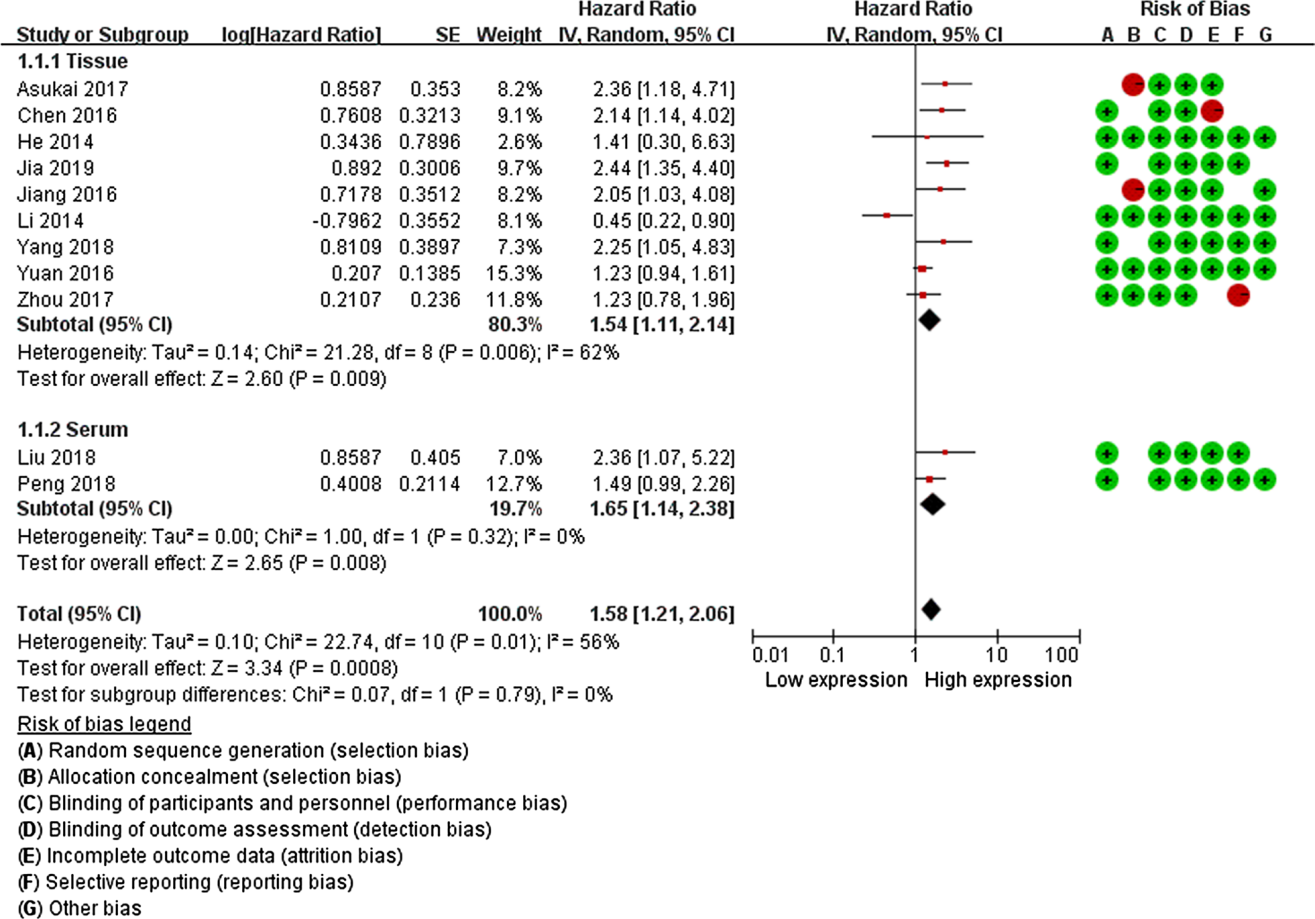
Forest plots for the relationship between miRNA-130a expression (tissue and serum) and overall survival (OS). The squares and horizontal lines correspond to the study-specific HR and 95% CI. The area of the squares reflects the study specific weight. The diamond represents the pooled HR and 95% CI

**Fig. 3:**
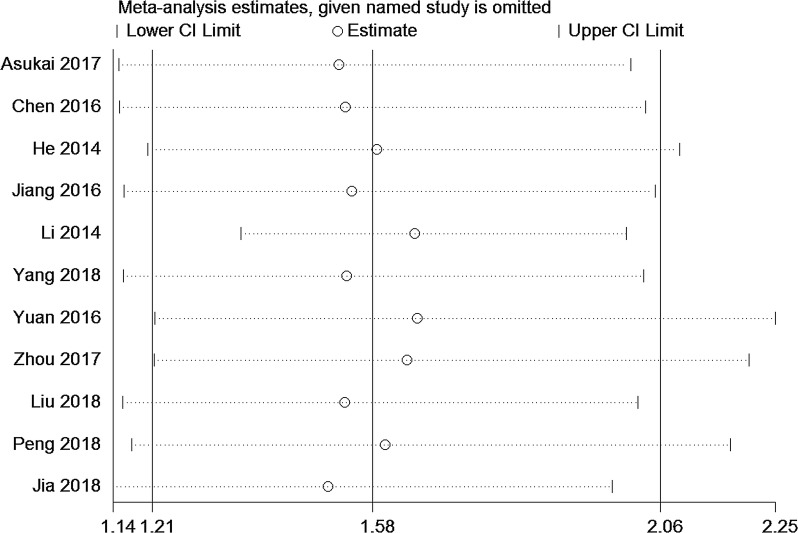
Sensitivity analysis for OS of miRNA-130a. Meta-analysis estimates, given named study is omitted for pooled results

Based on QUIPS, the quality assessment for eligible studies was presented in Table 2. The risk of bias legend was summarized in Figs. 2 and 3. According to the NOS (Additional file 1: Table S1), yielded scores ranging from 5 to 9, with a mean score of 6.63, 76.0% (19/25) of these studies were considered as high-quality.

**Table 2:** Quality assessment of included studies based on the Quality In Prognosis Studies (QUIPS)

Study [Ref.]	Quality evaluation of prognosis study						Total score^a^	Level of evidence^b^
	Study participation	Study attrition	Prognostic factor measurement	Outcome measurement	Study confounding	Statistical analysis and reporting		
Jia 2019 [44]	Yes	Partly	Yes	Yes	Partly	Yes	7	2b
Peng 2018 [18]	Yes	Partly	Yes	Yes	Partly	Yes	*7*	2b
Liu 2018 [45]	Yes	Partly	Partly	Yes	Partly	Yes	*5*	2b
Yang 2018 [46]	Yes	Partly	Yes	Yes	Partly	Yes	*7*	2b
Asukai 2017 [16]	Partly	Partly	Partly	Yes	Partly	Partly	*6*	2b
Zhou 2017 [47]	Yes	Partly	Yes	Partly	Partly	Partly	*7*	2b
Chen 2016 [11]	Yes	Partly	Yes	Partly	Partly	Partly	*5*	2b
Jiang 2016 [17]	Partly	Partly	Partly	Yes	Partly	Partly	5	2b
Yuan 2016 [14]	Yes	Partly	Yes	Yes	Partly	Yes	*8*	1b
He 2014 [15]	Yes	Yes	Yes	Yes	Partly	Yes	8	1b
Li 2014 [21]	Yes	Yes	Yes	Yes	Partly	Yes	7	2b
Wang 2012 [48]	Partly	Partly	Yes	Yes	Partly	Yes	6	2b
Hashimoto 2019 [49]	Yes	Yes	Yes	Yes	Partly	Yes	8	1b
Ulivi 2019 [50]	Partly	Partly	Yes	Yes	Partly	Yes	6	2b
Hu 2018 [51]	Yes	Yes	Yes	Partly	Partly	Partly	9	2b
Ecke 2017 [52]	Yes	Partly	Yes	Yes	Partly	Yes	7	2b
Li 2017 [22]	Yes	Partly	Yes	Yes	Partly	Yes	7	2b
Chang 2016 [23]	Yes	Yes	Yes	Partly	Partly	Partly	8	2b
Sheng 2015 [24]	Partly	Partly	Yes	Yes	Partly	Yes	7	2b
Kjersem 2014 [30]	Partly	Partly	Yes	Partly	Partly	Partly	6	2b
Wang 2014 [25]	Partly	Partly	Yes	Yes	Partly	Yes	7	2b
Colangelo 2013 [26]	Partly	Partly	Yes	Partly	Partly	Partly	5	2b
Zhao 2013 [31]	Partly	Partly	Yes	Yes	Partly	Yes	6	2b
Nakatani 2012 [53]	Partly	Partly	Partly	Yes	Partly	Yes	5	2b

^a^Quality assessment of included studies based on the Newcastle–Ottawa Scale

^b^The levels of evidence were estimated for all included studies with the Oxford Centre for Evidence Based Medicine criteria

### Quantitative synthesis

#### The expression of miRNA-130a and patients’ survival

The observed random effect size of pooled HRs for OS were provided by 10 studies, the results indicated that the high levels of miRNA-130a expression was correlated with poor OS in cancer patients (HR = 1.58, 95% CI: 1.21–2.06, *P *< 0.001). Tissue and serum expression of miRNA-130a was significantly correlated with the OS (HR = 1.54, 95% CI: 1.11–2.14, *P *= 0.009; HR = 1.65, 95% CI: 1.14–2.38, *P *= 0.008). For the subgroup differences test, the results indicated no heterogeneity between subgroups (^2^ = 0%, =0.79) (Table 3, Fig. 2).

**Table 3:** Main results of pooled HRs in the meta-analysis

Comparisons (miRNA-130 family)	Heterogeneity test			Summary HR (95% CI)	Hypothesis test		Studies
	*Q*	*P*	*I*^*2*^ (%)		*Z*	*P*	
miRNA-130a							
OS							
Total	22.74	0.01	56	1.58 (1.21,2.06)	3.34	< 0.001	11
Tissue	21.28	0.01	62	1.54 (1.11,2.14)	2.60	0.009	9
Serum	1.00	0.32	0	1.65 (1.14,2.38)	2.65	0.008	2
Subgroup differences	0.07	0.79	0				
Cancer subtypes							
Gastric cancer	1.94	0.38	0	1.81 (1.34,2.45)	3.83	< 0.001	3
Other cancers	18.30	0.01	62	1.46 (1.01,2.08)	2.11	0.03	8
DFS							
Total	24.08	< 0.01	79	1.35 (0.72,2.52)	0.93	0.35	6
Tissue	23.91	< 0.01	83	1.32 (0.52,3.40)	0.58	0.56	5
Serum	–	–	–	1.38 (0.99,1.91)	0.21	0.83	1
Subgroup differences	0.01	0.94	0				
miRNA**-**130b							
OS							
Total	60.10	< 0.01	77	1.95 (1.47,2.59)	4.65	< 0.001	15
Tissue	44.46	< 0.01	75	2.01 (1.39,2.91)	3.71	< 0.001	12
Serum	15.50	< 0.01	87	1.96 (1.09,3.54)	2.23	0.03	3
Subgroup differences	0.01	0.94	0				
Ethnicity							
Asian	12.15	0.06	51	2.55 (1.77,3.69)	5.00	< 0.001	7
Caucasian	23.95	< 0.01	71	1.47 (1.08,1.99)	2.45	0.01	8
Cancer subtypes							
HCC	10.59	0.01	72	2.43 (1.28,4.63)	8.24	0.004	4
Other cancers	30.80	< 0.01	74	1.75 (1.30,2.37)	3.67	< 0.001	11
DFS*/*PFS							
Total	7.38	0.12	46	1.53 (1.31,1.77)	5.53	< 0.001	5
Tissue	2.48	0.29	19	1.98 (1.50,2.62)	4.85	< 0.001	3
Serum	0.03	0.86	0	1.37 (1.15,1.64)	3.46	< 0.001	2
Subgroup differences	4.87	0.03	79.5				
Cancer subtypes							
HCC (DFS)	2.48	0.29	19	1.98 (1.50,2.62)	4.85	< 0.001	3
Other cancers	0.03	0.86	0	1.37 (1.15,1.64)	3.46	< 0.001	2

OS, overall survival; DFS, disease free survival; PFS, progressive free survival; HCC, hepatocellular carcinoma

According to cancer subtype, the subgroup analysis was performed, the results showed that high expression of miRNA-130a was significantly correlated with poor OS in gastric cancer (HR = 1.81, 95% CI: 1.34–2.45, *P *< 0.001) and other cancer types (HR = 1.46, 95% CI: 1.01–2.08, *P *= 0.03) (Table 3).

The pooled HRs for DFS were provided by 6 studies, there was no significant correlation between miRNA-130a expression and DFS (HR = 1.35, 95% CI: 0.72–2.52, *P *= 0.35), and tissue and serum expression of miRNA-130a was also not associated with the DFS (HR = 1.32, 95% CI: 0.52–3.40, *P *= 0.56; HR = 1.38, 95% CI: 0.99–1.91, *P *= 0.83) (Fig. 2, Table 3).

#### The expression of miRNA-130b and patients’ survival

The pooled HRs for OS were provided by 15 studies, there was a high correlation between high miRNA-130b expression and poor OS in cancer patients (HR = 1.95, 95% CI: 1.47–2.59, *P *< 0.001) (Fig. 3, Table 3). According to tissue and serum expression, the result indicated that a high expression of miRNA-130b significantly predicted pool OS in tissue sample (HR = 2.01, 95% CI: 1.39–2.91, *P *< 0.001) and serum sample (HR = 1.96, 95% CI: 1.09–3.54, *P *= 0.03) (Table 3). For the subgroup differences test, the results indicated that that there was heterogeneity between subgroups (^2^ = 0%, =0.94) (Table 3, Fig. 2).

Stratified analysis was performed based on ethnicity, the expression of miRNA-130b was significantly correlated with OS in Asian (HR = 2.55, 95% CI: 1.77–3.69, *P *< 0.001) and Caucasian (HR = 1.47, 95% CI: 1.08–1.99, *P *= 0.01). Subgroup analysis was also conducted according to cancer subtype, the expression of miRNA-130b was significantly associated with OS in HCC (HR = 2.43, 95% CI: 1.28–4.63, *P *= 0.004) and other cancer types (HR = 1.75, 95% CI: 1.30–2.37, *P *< 0.001) (Table 3).

Five studies provided a combined HR of disease progression, miRNA-130b expression was significantly associated with DFS/PFS (HR = 1.53, 95% CI: 1.31–1.77, *P *< 0.001). Furthermore, tissue and serum expression of miRNA-130b was significantly associated with the DFS/PFS (HR = 1.98, 95% CI: 1.50–2.62, *P *< 0.001; HR = 1.37, 95% CI: 1.15–1.64, *P *< 0.001). Test for subgroup differences, the results showed that there was a slight heterogeneity between subgroups (^2^ = 79.5%, =0.03) (Table 3).

According to cancer subtype, we conducted subgroup analysis, a significant association between increased miRNA-130b and poor DFS in patients with HCC (HR = 1.98, 95% CI: 1.50–2.62, *P *< 0.001), and DFS/PFS with other cancers (HR = 1.37, 95% CI: 1.15–6.64, *P *< 0.001).

### Test of heterogeneity

The heterogeneity of miRNA-130a for OS and miRNA-130b for subgroup differences was statistically significant (*P*_heterogeneity_ < 0.1 and *I*^2^ > 50%). Therefore, the random effects were used to calculate the HRs for miRNA-130a and miRNA-130b. Meanwhile, the meta-regression was used to explore the heterogeneous sources of miRNA-130a and miRNA-130b (Table 4).

**Table 4:** The results of heterogeneity test

Comparisons	Coef.	Std. Err.	*t*	*P*	95% CI
miRNA**-**130a					
Language	− 0.408	0.382	− 1.07	0.327	− 1.342 to 0.528
Publication year	− 1.272	0.734	− 1.73	0.134	− 3.069 to 0.525
Cancer type	0.549	0.710	0.77	0.469	− 1.188 to 2.287
Ethnic^a^	–	–	–	–	–
Assay^a^	–	–	–	–	–
Sample size	− 0.279	0.372	− 0.75	0.481	− 0.630 to 1.189
Follow-up	− 0.308	0.378	− 0.81	0.446	− 1.235 to 0.617
Cut-off	− 0.506	0.385	− 1.31	0.237	− 1.447 to 0.436
miRNA**-**130b					
Language^a^	–	–	–	–	–
Publication year	0.306	0.458	0.67	0.523	− 0.751 to 1.362
Cancer type	− 0.001	0. 542	− 0.00	0.999	− 1.250 to 1.248
Ethnic	− 0.678	0.606	− 112	0.296	− 2.076 to 0.720
Assay^a^	–	–	–	–	–
Sample size	0.107	0.568	0.19	0.856	− 1.202 to 1.416
Follow-up	− 0.169	0.600	− 0.28	0.786	− 1.552 to 1.215
Cut-off	0.178	0.318	0.56	0.591	− 0.556 to 0.912

^a^ Ethnic, language and assay were dropped because of collinearity

### Sensitivity analyses

To explore the stability of our results, sensitivity analysis was performed by removing one study at a time and recalculating pooled HR. The results (e.g. HR = 1.58, 95% CI: 1.21–2.06) did not substantially alter the combined HRs, which indicates that our results were quite robust and stable (Fig. 3).

### Assessment of publication bias

**Fig. 4:**
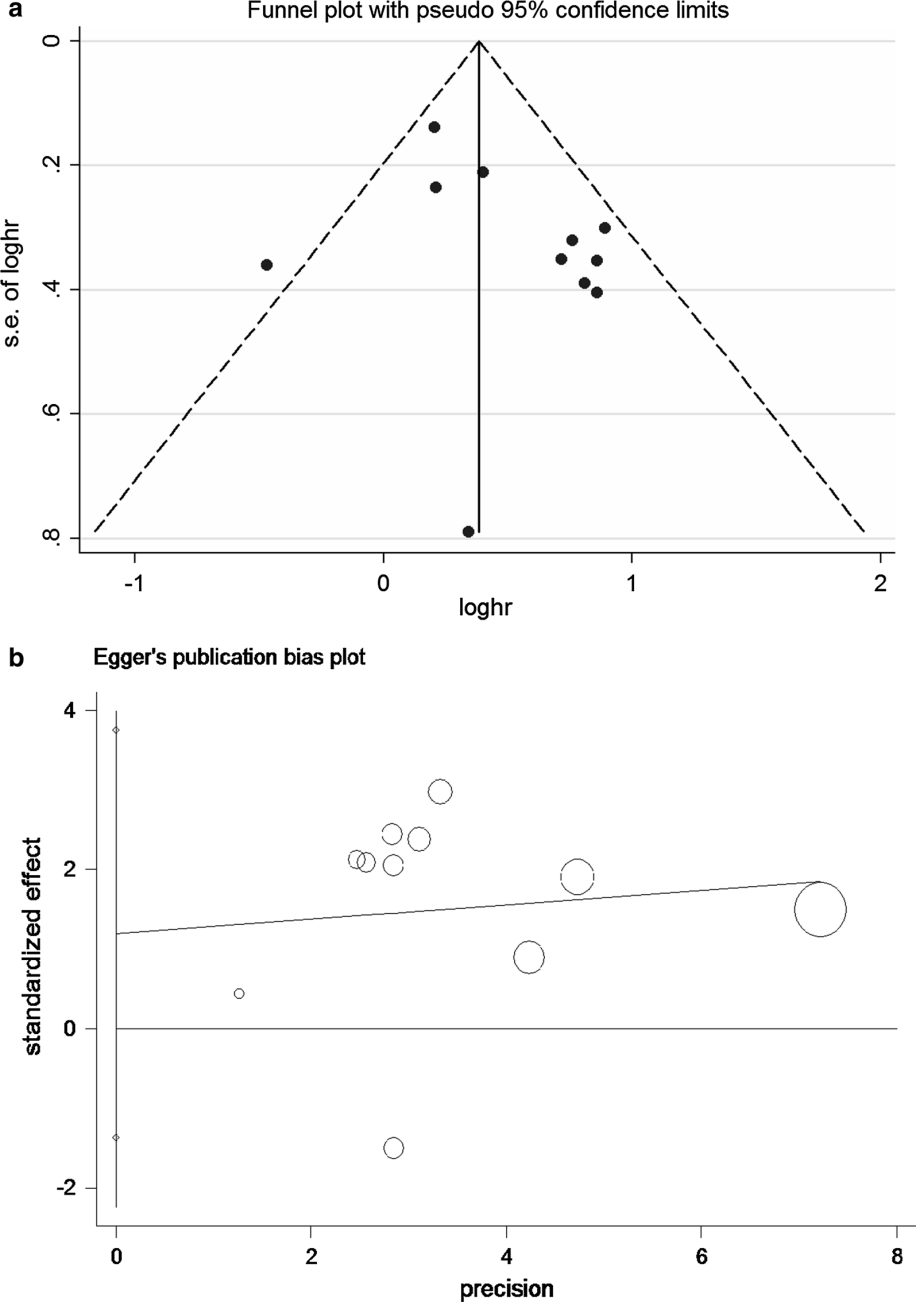
**a** Begg’s funnel plot of publication bias for the association between miRNA-130a expression and OS. The vertical line in the funnel plot indicates the fixed-effects summary estimate, whereas the sloping lines indicate the expected 95% confidence intervals for a given SE. **b** Egger’s test of publication bias for the association between miRNA-130a expression and OS. The horizontal line in the funnel plot indicates the fixed-effects summary estimate, whereas the sloping lines indicate the expected 95% confidence intervals for a given SE

Begg’s and Egger’s test were applied to evaluate the publication bias. The results didn’t reveal any evidence of publication bias (Additional file 2: Table S2). Meanwhile, the funnel plots shape was basically symmetrical (Fig. 4a, b).

### Expression of miRNA-130b and prognosis in database test

**Fig. 5:**
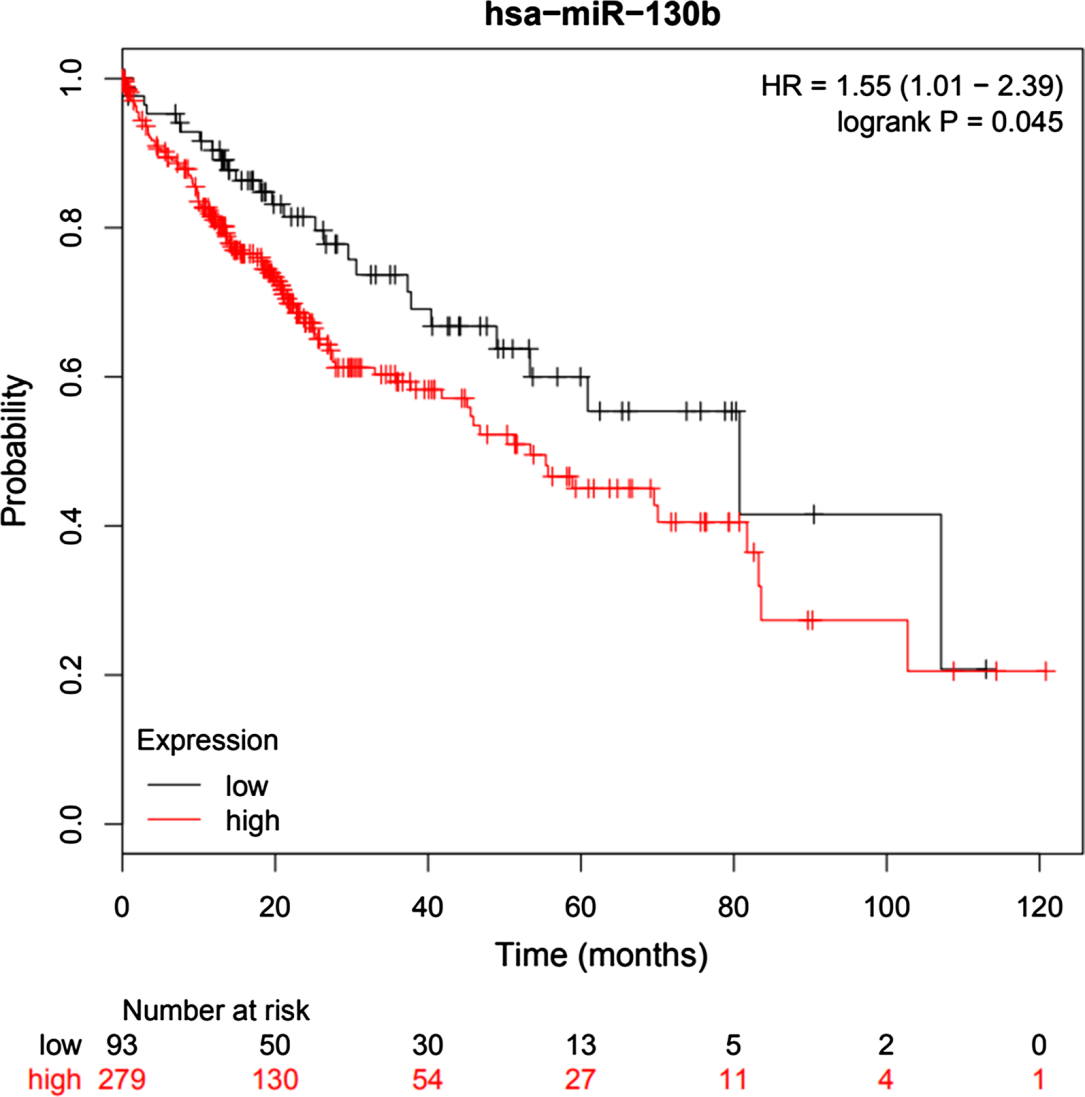
Kaplan–Meier survival curves for OS according to miRNA-130b expression in patients with HCC. OS of patients with high vs. low MMP-14 expression are shown

For OS, a highly significant correlation was revealed between high miRNA-130b expression and poor OS (HR = 1.55, *P *= 0.045) in patients with HCC (Fig. 5). The results of direct sequencing and expression of the miRNA-130b are very close to those of our combined result of individual studies.

## Discussion

Recently, accumulating evidence has demonstrated that miRNAs play an important role in cancer progression, including differentiation, proliferation, metastasis and apoptosis, and act as oncogenes or tumor suppressors [54–56]. Therefore, exploring the miRNAs involved in tumorigenesis and their target genes may contribute to understanding the underlying mechanisms of cancer patients and provide valuable clues for the early prognosis of cancer [57, 58]. The previous studies have revealed that the presence of miRNAs in the circulation may as valuable diagnostic and prognostic biomarkers in some types of cancer [59, 60].

The latest advances in biomedical research facilitated the identification of a variety of molecular biomarkers that benefit cancer screening and detection, guide drug discovery, and improve survival depending on customized treatment [61]. Recently, many biomarkers have been identified that can provide important prognostic information. Nevertheless, this study is the first one to focus on the correlation between miRNA-130 family and prognosis in cancer, involving 2141 patients with OS and 1159 patients with DFS/PFS. The results provided by 12 eligible studies for miRNA-130a and 15 eligible studies for miRNA-130b, which increased expression of the miRNA-130 family predicted reduced survival in patients with cancer, indicating the prognostic value of miRNA-130 family.

The subgroup analysis indicated a closer relationship between elevated miRNA-130b expression and poor survival in the Asians (HR = 2.55, 95% CI: 1.77, 3.69). The reason may be that different dietary habits and heritage backgrounds between Asian and Western populations contribute to different survival outcomes and cancer histopathology. Among 27 studies, OS was reported in 15 types of cancer. The result revealed that increased miRNA-130 family yielded worse OS in gastric cancer (HR = 1.81, 95% CI: 1.34, 2.45 for miRNA-130a) and HCC (HR = 2.43, 95% CI: 1.28, 4.63 for miRNA-130b). However, further studies are needed to explore whether the pathological types of some cancers affect the prognosis of the miRNA130 family. Due to the included studies applied different indices to evaluate cancer progression, such as DFS and PFS, we pooled the indices to assess the prognostic significance of miRNA-130 family. The results indicated a subtle correlation between high miR-130 family expression and DFS/PFS for miRNA-130b (HR = 1.53, 95% CI: 1.31, 1.77), indicating that high miRNA-130a expression may be a favorable prognostic factor in DFS/PFS, especially in HCC (DFS) (HR = 1.98, 95% CI: 1.50, 2.62). However, further study is needed to confirm the role of miRNA-130 family in predicting the prognosis of different types of cancer.

In order to be able to infer reliable results, we chose to the inclusion of three studies on HCC and explored the prognostic value of miRNA-130b in GEO, EGA and TCGA, and then verified our results. We used the 372 paired HCC and normal tissues from the databases with OS and DFS data, the expression of miRNA-130b was significantly higher than that in normal control group. All these findings are further confirmed our conclusion, and indicated that miRNA-130b was validated as an independent prognostic factor for OS in HCC patients.

Invasion and migration are substantive processes for cells, and miRNA-130a has been shown to regulate invasive activities and metastatic in cancer cells [62]. Rab5a has been shown to be involved in cellular functions as an oncogene, and overexpression of miRNA-130a inhibits proliferation and apoptosis of breast cancer cells through Rab5a targeting [63]. In addition, miRNA-130a was up-regulated in gemcitabine-resistant clones of human cholangiocarcinoma cell lines, which indicated that absolute expression of miRNA-130a correlated with the viability of cholangiocarcinoma cells. The high miRNA-130a expression in the specimens of cholangiocarcinoma was closely related to poor prognosis [16]. Similar result was reported by Wang et al. [64], the findings indicated that the expression of miRNA-130A is correlated with tumor recurrence or distant organ metastasis and can be used for clinical prognosis and early diagnosis of patients with breast cancer.

The published studies have shown that miRNA-130b has a variety of biological functions, including promoting mesenchymal stem cells aging of bladder and colorectal cancer [6, 65], enhancing drug resistance of ovarian cancer cells, enhancing cell motility and downregulating thyroid hormones [66]. The miRNA-130b dysregulation are related to properties and many biological properties, the possible molecular mechanism and function of miRNA-130b inglioma cells, compared with the level of normal tissues of certain cancer types, with the increase of histological grade of glioma, the expression of microRNA-130b increased significantly [24]. Recently, miRNA-130b regulates the differentiation and proliferation of embryonic neural progenitor cells by targeting the X-linked fragile X mental retardation 1 gene [67], it has been identified as a reliable biomarker of glioma with significant prognostic value [29], our study bear out this experimental results. Thus, our results demonstrated that miRNA130 family may promote cancer growth with prognostic signifcance and can potentially be used as a novel drug target in the future.

Although our study is robust, there were some limitations. Firstly, because not all included studies provide the multivariate adjusted HRs, part of the HRs and 95% CI extracted from the survival curve. These calculated might be generated several tiny errors. Secondly, the cut-off values of included studies were used to assess the different miRNA-130 family expression, the true values may be different because of different algorithms. Thirdly, although there was no statistical evidence of publication bias, most eligible studies are in English, which may lead to publication bias. Finally, data on miRNA expression are obtained by different qRT-PCR methods (i.e. via SYBR green or TaqMan) or normalised upon different endogenous markers, which may influence the variation in results. In spite of above limitations, this meta-analysis about association between miRNA-130 family and cancer prognosis is certainly warranted.

## Conclusion

In summary, the high expression of the miRNA-130 family is significantly associated with poor survival in cancer patients, especially in gastric cancer and HCC. In addition, expression of miRNA-130b was associated with ethnicity, especially in Asian. Our findings suggested that the high expression of miRNA-130 family may be applied as a prognostic predictor in patients with cancer. Prospectively, combining miRNA-130a and miRNA-130b may be considered as powerful prognostic predictor for clinical application.

## Supplementary information

**Additional file 1: Table S1.** Quality assessment of included studies based on the Newcastle–Ottawa Scale for assessing the quality of cohort studies.

**Additional file 2: Table S2** Publication bias of miRNA-130 family for Begg’s test and Egger’s test.

## Data Availability

Not applicable.

## Abbreviations

HR: hazard ratios
95% CI: confidence intervals
OS: overall survival
DFS: disease-free survival
PSF: progression-free survival
NOS: Newcastle–Ottawa Scale (NOS)
QUIPS: the specific Quality In Prognosis Studies
qRT-PCR: quantitative real-time polymerase chain reaction
DTC: digestive tract cancer
HCC: hepatocellular cancer
