# Supplementary material for: Prognostic values of microRNA-130 family expression in patients with cancer: a meta-analysis and database test
Source: J Transl Med. 2019 Oct 22;17:347. doi: 10.1186/s12967-019-2093-y (PMC6805372; doi:10.1186/s12967-019-2093-y)
Supplement: Supplementary file 1 — Additional file 1: Table S1. Quality assessment of included studies based on the Newcastle–Ottawa Scale for assessing the quality of cohort studies. [file 12967_2019_2093_MOESM1_ESM.docx]

**Table S1. Quality assessment of included studies based on the Newcastle–Ottawa Scale for assessing the quality of cohort studies.**

| Study [Ref.] | Selection  (score) |  |  |  |  | Comparability  (score) |  | Exposure  (score) |  |  |  |
| --- | --- | --- | --- | --- | --- | --- | --- | --- | --- | --- | --- |
|  | Representativenes  of the exposed cohort | Selection of the  non-exposed cohort | Ascertainment  of exposure | Outcome of interest was not present at  start of study |  | Based on the  design or analysis ^a^ |  | Assessment  of outcome | Follow-up long  enough for  outcomes to occur | Adequacy of  follow-up  of cohorts | Total  Score^b^ |
| Jia 2019 [44] | 1 | 0 | 1 | 1 |  | 2 |  | 1 | 1 | 0 | **7** |
| Peng 2018 [18] | 1 | 0 | 1 | 1 |  | 2 |  | 1 | 1 | 0 | **7** |
| Liu 2018 [45] | 1 | 0 | 1 | 1 |  | 0 |  | 1 | 1 | 0 | **5** |
| Yang 2018 [46] | 1 | 0 | 1 | 1 |  | 2 |  | 1 | 1 | 0 | **7** |
| Asukai 2017 [16] | 1 | 0 | 1 | 1 |  | 2 |  | 1 | 1 | 0 | **6** |
| Zhou 2017 [47] | 1 | 0 | 1 | 1 |  | 2 |  | 1 | 1 | 0 | **7** |
| Chen 2016 [11] | 1 | 0 | 1 | 1 |  | 2 |  | 1 | 1 | 0 | **5** |
| Jiang 2016 [17] | 1 | 0 | 1 | 1 |  | 0 |  | 1 | 1 | 0 | **5** |
| Yuan 2016 [14] | 1 | 0 | 1 | 1 |  | 2 |  | 1 | 1 | 1 | **8** |
| He 2014 [15] | 1 | 0 | 1 | 1 |  | 2 |  | 1 | 1 | 1 | **8** |
| Li 2014 [21] | 1 | 0 | 1 | 1 |  | 1 |  | 1 | 1 | 1 | **7** |
| Wang 2012 [48] | 1 | 0 | 0 | 1 |  | 2 |  | 1 | 1 | 0 | **6** |
| Hashimoto 2019 [49] | 1 | 1 | 1 | 1 |  | 1 |  | 1 | 1 | 1 | **8** |
| Ulivi,2019 [50] | 1 | 0 | 1 | 1 |  | 1 |  | 1 | 1 | 0 | **6** |
| Hu 2018 [51] | 1 | 1 | 1 | 1 |  | 2 |  | 1 | 1 | 1 | **9** |
| Ecke 2017 [52] | 1 | 0 | 1 | 1 |  | 2 |  | 1 | 1 | 0 | **7** |
| Li 2017 [22] | 1 | 0 | 1 | 1 |  | 2 |  | 1 | 1 | 0 | **7** |
| Chang 2016 [23] | 1 | 1 | 1 | 1 |  | 2 |  | 1 | 1 | 0 | **8** |
| Sheng 2015 [24] | 1 | 0 | 1 | 1 |  | 2 |  | 1 | 1 | 0 | **7** |
| Wang 2014 [25] | 1 | 0 | 1 | 1 |  | 2 |  | 1 | 1 | 0 | **7** |
| Kjersem 2014 [30] | 1 | 0 | 0 | 1 |  | 2 |  | 1 | 1 | 0 | **6** |
| Colangelo 2013[26] | 1 | 0 | 0 | 1 |  | 2 |  | 0 | 1 | 0 | **5** |
| Zhao 2013 [31] | 1 | 0 | 0 | 1 |  | 2 |  | 1 | 1 | 0 | **6** |
| Nakatani 2012 [53] | 1 | 0 | 1 | 1 |  | 0 |  | 1 | 1 | 0 | **5** |

^a^ When there was no statistical significance in the response rate between case and control groups by using a chi-squared test (*P* > 0.05), one point was awarded.

^b^Total score was calculated by adding up the points awarded in each item.
