# Supplementary material for: Prognostic values of microRNA-130 family expression in patients with cancer: a meta-analysis and database test
Source: J Transl Med. 2019 Oct 22;17:347. doi: 10.1186/s12967-019-2093-y (PMC6805372; doi:10.1186/s12967-019-2093-y)
Supplement: Supplementary file 2 — Additional file 2: Table S2 Publication bias of miRNA-130 family for Begg’s test and Egger’s test. [file 12967_2019_2093_MOESM2_ESM.docx]

**Table S2 Publication bias of miRNA-130 family for Begg’s test and Egger’s test.**

| **Comparisons** | Begg’s test | |  | Egger’s test | | |
| --- | --- | --- | --- | --- | --- | --- |
|  | *z* | *p* |  | *t* | *p* | 95% CI |
| **miRNA-130a** |  |  |  |  |  |  |
| OS | 0.18 | 0.855 |  | 0.67 | 0.518 | -0.594-1.111 |
| DFS | 0.38 | 0.707 |  | -0.22 | 0.839 | -6.222-5.319 |
| **miRNA-130b** |  |  |  |  |  |  |
| OS | 1.83 | 0.064 |  | 1.79 | 0.121 | -0.813-3.924 |
| DFS/PFS | 1.91 | 0.079 |  | 2.32 | 0.101 | -0.639-5.890 |
